# Individual variation in the vigor and form of Pavlovian conditioned responses: Analysis of a model system

**DOI:** 10.1016/j.lmot.2020.101658

**Published:** 2020-11

**Authors:** Robert C. Honey, Dominic M. Dwyer, Adela F. Iliescu

**Affiliations:** Cardiff University, UK

**Keywords:** Pavlovian conditioning, HeiDI, Individual differences

## Abstract

Pavlovian conditioning results in individual variation in the vigor and form of acquired behaviors. Here, we describe a general-process model of associative learning (HeiDI; How excitation and inhibition determine ideo-motion) that provides an analysis for such variation together with a range of other important group-level phenomena. The model takes as its starting point the idea that pairings of a conditioned stimulus (CS) and an unconditioned stimulus (US) result in the formation of reciprocal associations between their central representations. The asymptotic values of these associations and the rate at which these are reached are held to be influenced by the perceived salience of the CS (α_CS_) and US (β_US_). Importantly, whether this associative knowledge is exhibited in behavior that reflects the properties of the CS (e.g., sign-tracking) or US (e.g., goal-tracking) is also influenced by the relative values of α_CS_ and β_US_. In this way, HeiDI provides an analysis for both quantitative and qualitative individual differences generated by Pavlovian conditioning procedures.

“*Give me a dozen healthy infants, well-formed, and my own specified world to bring them up in and I'll guarantee to take any one at random and train him to become any type of specialist I might select – doctor, lawyer, artist, merchant-chief and, yes, even beggar-man and thief, regardless of his talents, penchants, tendencies, abilities, vocations, and race of his ancestors. I am going beyond my facts, and I admit it, but so have the advocates to the contrary, and they have been doing so for many thousands of years*.” (p. 82; Watson, 1924).

Watson’s central thesis might seem less controversial now than it did almost a century ago: An appropriately conducive environment – where different forms of training can be arranged – affords the development of selected paths in infants taken randomly from the normal population. His central thesis did not deny the existence of individual differences that have different origins (e.g., talents, penchants etc.), but it did suggest that training might free an individual from them. However, the fact that dramatic individual differences in acquired behavior can emerge in animals given identical training in a controlled environment is – if not antithetical – then certainly problematic from an empiricist perspective (e.g., Iliescu, Hall, Wilkinson, Dwyer, & Honey, 2018; Patitucci, Nelson, Dwyer, & Honey, 2016; but see, Byrom & Murphy, 2018). For example, such individual variation is beyond the scope of general-process theories of associative learning (e.g., Rescorla & Wagner, 1972; Mackintosh, 1975; Pearce & Hall, 1980; Wagner, 1981) in which the relationship between the strength of an association and performance is held to be monotonic: How could a single acquired property (like associative strength; V) be manifest in distinct ways across a set of rats? We have recently presented a model, HeiDI, which offers a potential answer to this question (Honey et al., 2020a, 2020b). Before we present that answer, we should first describe results that provided an important impetus for the development of HeiDI.

## 1 Individual differences in conditioned behavior

**Fig. 1:**
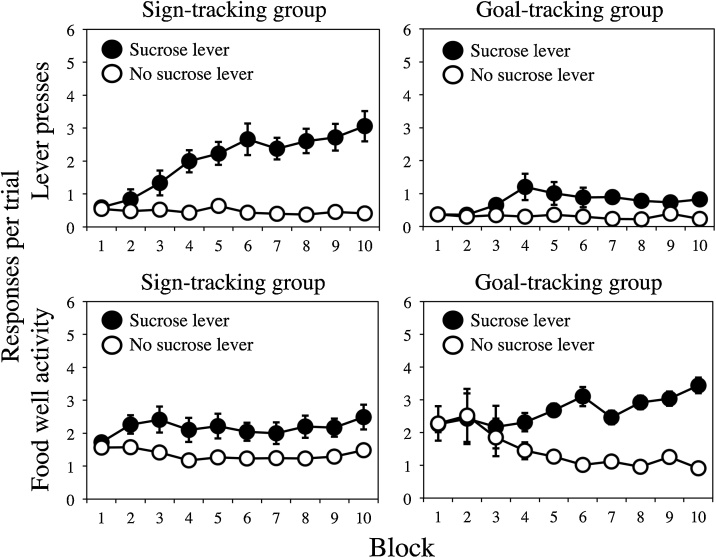
The emergence of qualitative differences in conditioned behavior across 10 blocks of training. Mean (± SEM) levels of lever activity (sign-tracking) and food well activity (goal-tracking). Rats were divided into sign-trackers (left panels) and goal-trackers (right panels), with scores separated for the lever paired with sucrose and the lever that was not. Adapted from: Patitucci, E., Nelson, N., Dwyer, D.M., & Honey, R.C. (2016). The origins of individual differences in how learning is expressed in rats: A general-process perspective. *Journal of Experimental Psychology*: *Animal Learning and Cognition, 42,* 313-324.

What happens when a given set of healthy rats is food restricted and then receives identical training trials in a standard conditioning chamber? If the temporary insertion of a lever into the chamber serves as the conditioned stimulus (CS) and the delivery of sucrose (for example) serves as the unconditioned stimulus (US), then the CS will come to elicit conditioned responses (CRs): The rats develop a tendency to interact with the lever and to approach the well into which sucrose is about to be delivered. The fact that the CS can provoke multiple CRs is of interest in its own right: It suggests that the CR is not simply determined by the unconditioned responses provoked by the US (e.g., to approach the location where the US will be delivered; cf. Pavlov, 1927). More interesting, however, is the fact that the distribution of these two forms of CR differ across rats given this autoshaping procedure: with some being more likely to interact with the lever (called sign-tracking; e.g., Hearst & Jenkins, 1974) than to approach the food well (called goal-tracking; e.g., Boakes, 1977), and others being more likely to approach the food well than to interact with the lever (e.g., Iliescu et al., 2018; Patitucci et al., 2016). This variation is relatively continuous in nature and remarkably stable from one day to the next: These are *qualitative* individual differences in acquired behavior (see Fitzpatrick et al., 2013; see also, Matzel et al., 2003). Fig. 1 illustrates the results of a representative experiment in which rats received training where the insertion of one lever (for 10 s) was followed by the delivery of sucrose, while the insertion of another was not (Experiment 2; Patitucci et al., 2016). The rats have been separated into two groups based on their bias to sign-track and goal-track in the final block of training; with the bias scores calculated in the following way: (number goal-tracking responses – number of sign-tracking responses) / (number goal-tracking responses + number of sign-tracking responses). Using this measure of bias to split the rats into two groups enables the development of sign-tracking and goal-tracking behaviors to be illustrated. But as already noted, the individual differences are relatively continuous across the group of rats as a whole, as we will have cause to return to at a later point.

It takes a moment to fully appreciate the theoretical significance of the fact that rats given identical training exhibit what they have learnt in qualitatively different ways: There is no coherent mapping between learning and the two measures of performance (sign-tracking and goal-tracking). Focusing on lever-oriented behavior suggests that the sign-tracking group learnt more readily than the goal-tracking group, while focusing on food-well oriented behavior suggests just the opposite. This is true whether one construes learning as the development of associations between the representation of the CS (the lever) and the processes responsible for generating responding (e.g., Hull, 1943; Spence, 1937) or between the representations of the CS and the US (e.g., Mackintosh, 1975; Pearce & Hall, 1980; Rescorla & Wagner, 1972). Indeed the problem is yet more ubiquitous: It is a challenge to any model that assumes that a single process underlies learning and its translation into performance (e.g., Gallistel & Gibbon, 2000; Stout & Miller, 2007). In the context of associative theories of learning, the problem reflects a surprising reluctance to specify how the strength of a CS-US association (i.e., V_CS-US_) is translated into conditioned responding: with most models simply assuming that there is a monotonic relationship between V_CS-US_ and conditioned behavior. What is required is a general-process model that (i) specifies the mnemonic structures that underpin learning and performance, (ii) describes the rules governing how change occurs within those structures, and (iii) isolates potential sources of individual differences. HeiDI is such a model.

## 2 HeiDI

**Fig. 2:**
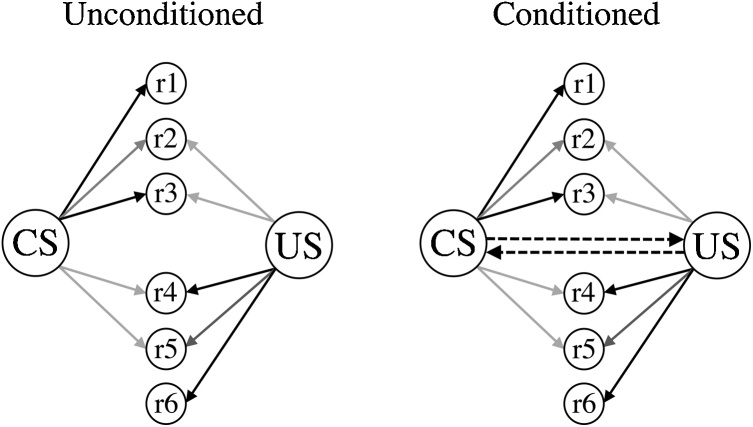
A schematic of the associative structures that are assumed to underpin Pavlovian learning and performance. The left-hand depicts the unconditioned structure (i.e., before conditioning), with the darkness of the links between the CS and r1-r6 and the US and r1-r6 indicating their strength; and the right-hand side depicts the conditioned structure (i.e., after conditioning). The reciprocal CS-US and US-CS associations are denoted by the dashed lines. Adapted from: Honey, R.C., Dwyer, D.M., & Iliescu, A.F. (2020). HeiDI: A model for Pavlovian learning and performance with reciprocal associations. *Psychological Review* (in press).

The mnemonic structures assumed to underpin learning and performance are depicted in Fig. 2. The left-hand side shows the pattern of unconditioned links between the CS, US and a set of response generating units (r1-r6). Some of these units are more strongly activated by the CS than the US (r1-r3), and some are more strongly activated by the US than the CS (r4-r6), with the darkness of the links denoting their strength. In this way, a general distinction is made between CS-oriented behaviors (r1-r3; e.g., orienting, lever approach, rearing) and US-oriented behaviors (r4-r6; e.g., food well approach, chewing, swallowing; see also, Holland, 1977, 1984). The right-hand side of Fig. 2 shows the conditioned structure, where standard Pavlovian conditioning trials – in which the CS immediately precedes the US – are assumed to result in the formation of reciprocal (CS-US and US-CS) associations (cf. Asratian, 1952, 1965; Pavlov, 1932; for a review, see Goremezano & Tait, 1976; but see, Konorski, 1948). In this way, a minimal functional cell assembly is formed: When the CS is presented activation propagates to the US, which is propagated back to the CS (e.g., Grossberg, 1980; Hebb, 1949). This structure contrasts with other trial-based models of associative learning (e.g., Mackintosh, 1975; Pearce & Hall, 1980; Rescorla & Wagner, 1972) where the focus has been on the formation of CS-US associations, with their functional alignment to a process of prediction in spite of the fact that the models are trial based (i.e., the change in associative strength is assumed to occur upon presentation of the US). The reciprocal nature of the associations within HeiDI means that there is a basis for CS-oriented conditioned responses to change, through the influence of the US-CS association, independently of the influence of the CS-US association, which primarily affects US-oriented conditioned responses. The rules governing the formation of these associations are captured in Eqs. 1 and 2. Eq. 1 determines the development of the CS-US association, and is a simplification to the Rescorla-Wagner error-correcting learning rule, while Eq. 2 is the formally equivalent rule for the development of US-CS associations. These equations both reduce the number of free parameters from those in the Rescorla-Wagner model, and generate increased explanatory power, especially when coupled with formally equivalent equations for determining the formation of associations between two CSs (which we will come to shortly).

(1) $$\Delta {\text{V}}_{\text{CS-US}}={\text{α}}_{\text{CS}}\left(\text{c}\text{.}{\text{β}}_{\text{US}}-\Sigma {\text{V}}_{\text{TOTAL-US}}\right)$$

(2) $$\Delta {\text{V}}_{\text{US-CS}}={\text{β}}_{\text{US}}\left(\text{c}\text{.}{\text{α}}_{\text{CS}}-\Sigma {\text{V}}_{\text{TOTAL-CS}}\right)$$

The perceived salience of the US determines the asymptote for the CS-US association, and at the same time the perceived salience of the CS determines the asymptote for the US-CS association. In Eq. 1, the maximum strength of the CS-US association is 1 in units of V (denoted c) modulated by the value of the parameter β_US_, which aligns to the perceived salience of the US (i.e., c.β_US_). The value of α_CS_ determines the rate at which the CS-US association changes, and aligns to the perceived salience of the CS. In Eq. 2, the maximum strength of the US-CS association is again 1 in units of V (i.e., c), and is modulated by the value of α_CS_ (i.e., c.α_CS_). In this case, the value of β_US_ determines the rate at which the US-CS association changes. α_CS_ and β_US_ are confined to the unit interval 0≤ α_CS_, β_US_ ≤ 1, and thus modulate both the rate of learning and the maximum strength of the reciprocal associations. In Eqs. 1 and 2, when the CS is absent, α_CS_ and c.α_CS_ are both set to 0 and when the US is absent, β_US_ and c.β_US_ are both set to 0. This arrangement allows the strength of the CS-US association to change on trials on which the CS is present, but the US is no longer presented, and the US-CS association to change on trials on which the US is presented but the CS is absent (cf. Wagner & Rescorla, 1972; Wagner, Logan, Haberlandt, & Price, 1968).

According to Eq. 1, during simple conditioning, the strength of the CS-US association (i.e., V_CS-US_) converges asymptotically on c.β_US_, with the change in associative strength of this association on a given trial (ΔV_CS-US_) being determined by the error in the pooled error term for the US (c.β_US_ – ΣV_TOTAL-US_). ΣV_TOTAL-US_ denotes the total net associative strength of the CS with respect to the US, and excitatory learning stops when ΣV_TOTAL-US_ = c.β_US_, and the learning rate parameter α_CS_ affects the rate at which V_CS-US_ approaches c.β_US_. Under conditions in which more than one CS (e.g., stimulus A and stimulus B) is paired with a US, the pooled error term means that the development of the A-US association will be influenced by the strength of the B-US association. That is, ΣV_TOTAL-US_ is equal to the total or combined associative strengths of A and B with respect to the US.

Eq. 2 is the complementary rule governing the formation of the US-CS association. Here, the change in the strength of this association (ΔV_US-CS_) on a given trial is also determined by the error within the pooled error term for the CS (c.α_CS_ – ΣV_TOTAL-CS_); with ΣV_TOTAL-CS_ denoting the associative strength of the single US (in typical conditioning procedures) with respect to the CS. Learning ceases when ΣV_TOTAL-CS_ = c.α_CS_, and the learning rate parameter β_US_ affects the rate at which V_US-CS_ approaches c.α_CS_. If we now consider what happens on a trial in which a compound of two stimuli (A and B) precedes a US, then the c.α_CS_ values for each CS in a compound (i.e., c.α_A_ and c.α_B_) set independent asymptotes for the US-A and US-B associations; and B will compete with the US for association with A, and A will compete with the US for association with B (see Honey et al., 2020a, 2020b).

The associative structures depicted in Fig. 2, together with the learning (Eqs. (1), (2), (3) and performance (Eqs. 4 and 5) rules, are readily extended to the case where two CSs (A and B) become linked. In Eqs. 1 and 2, for example, α_CS_ and β_US_ can be replaced with α_A_ and α_B,_ respectively. In fact, group-level differences in such associations enable HeiDI to explain some of the phenomena that were beyond the scope of the model proposed by Rescorla and Wagner (1972); see Honey et al. (2020a, 2020b). But, individual differences in the perceived saliences of A and B could also constitute a basis for individual differences in learning and performance. These differences could be directly observed during pairings of two stimuli that evoke different unconditioned behaviors (e.g., a tone with a light; see Honey, Good, & Manser, 1998; Honey, Watt, & Good, 1998; see also, Narbutovich & Podkopayev, 1936; cited in Konorski, 1948, p. 91), or indirectly observed through a range of sensory preconditioning procedures (e.g., Pavlov, 1931/1932; cited in Kimmel, 1977; see also Brogden, 1939; Rescorla & Cunningham, 1978; Ward-Robinson & Hall, 1996). However, as far as we are aware, there have been no studies that have examined whether or not such individual differences are evident in the vigor or form of conditioned behavior acquired as a consequence of sensory-sensory pairings.

We have already noted that HeiDI assumes that α_CS_ and β_US_ are aligned to the perceived salience of the CS and US, respectively. When this assumption is combined with the learning rules (i.e., Eqs. 1 and 2) it is clear that individual differences in the perceived salience of the CS and US will affect both the asymptotic values of the CS-US and US-CS associations, and the rates at which these values are reached. That is, *quantitative* differences in learning are predicted to the extent that there are differences in the perceived salience of the CS and US. However, the individual differences in CS-oriented and US-oriented conditioned behaviors (e.g., sign-tracking and goal-tracking) are not only quantitative but also *qualitative* (see Fig. 1). This fact clearly requires that there is a more complex mapping of associative strength than a monotonic one. HeiDI first assumes that upon presentation of the CS, the associative strengths returned by Eqs. 1 and 2 are combined (V_COMB_) in the way specified in Eq. 3. V_COMB_ represents the associative resonance within the CS-US assembly, and Eq. 3 weights the associative strength of the stimulus that is present (e.g., V_CS-US_) more than the association involving an associatively activated node (e.g., V_US-CS_; see Equation 3). In Eq. 3, V_COMB_ is in units of V, because V_CS-US_ is rendered dimensionless by multiplying it by 1/c. This combination rule captures the idea that the two stimuli function as an assembly, but one in which associative activity generated by the presentation of a stimulus (e.g., the CS) is subject to a process of dampening. That is, the US-CS association is only activated to the degree that the US itself is activated via the CS-US association.

(3) $${\text{V}}_{\text{COMB}}={\text{V}}_{\text{CS-US}}+\left(\frac{1}{\text{c}}.{\text{V}}_{\text{CS-US}}\times {\text{V}}_{\text{US-CS}}\right)$$

Critically, HeiDI assumes that when the CS is presented, V_COMB_ is distributed into two components, which have different influences on performance: A CS-oriented component (which influences sign-tracking), and a US-oriented component (which influences goal-tracking). One way to do this, is according to the relative perceived saliences of the CS and US (i.e., α_CS_ and β_US_): If α_CS_ > β_US_ then CS-oriented behavior would dominate US-oriented behavior, and if β_US_ > α_CS_ then the reverse is the case. However, this proposal is inadequate, because while the perceived salience of a CS will be available upon CS presentation, the perceived salience of the US will not: The US is not present. We have proposed, therefore, that the distribution of V_COMB_ is determined by the value of α_CS_ relative to V_CS-US_ (which reflects β_US_). That is, HeiDI assumes that V_COMB_ is distributed into the CS-oriented and US-oriented components according to the perceived salience of the CS (α_CS_) relative to the perceived salience of the retrieved US representation (i.e., V_CS-US_); which is indirectly influenced by the perceived salience of the US (i.e., β_US_; see Eq. 1). This idea is formally expressed in Eqs. 4 and 5 that generate two components, R_CS_ and R_US_, which are held to affect the levels of CS-oriented and US-oriented responding, respectively. According to these equations, when α_CS_ > V_CS-US_ then R_CS_ > R_US_, but the reverse is the case when V_CS-US_ > α_CS_. That is, the balance between CS- and US-oriented behavior is related to the individually perceived salience of the CS and US themselves. To address the fact that Eq. 1 (and Eq. 2) can return negative Vs, the absolute value of V_CS-US_ is used in Eqs. 4 and 5 to ensure that the proportions are < 1. As before, |V_CS-US_| is transformed into a dimensionless value by multiplying it by 1/c, which means that R_CS_ and R_US_ assume units of V. Clearly, Eqs. 4 and 5 are intimately related, being reciprocals of one another.

(4) $${\text{R}}_{\text{CS}}=\frac{{\text{α}}_{\text{CS}}}{{\text{α}}_{\text{CS}}+\frac{1}{\text{c}}.\left|{\text{V}}_{\text{CS-US}}\right|}{\text{V}}_{\text{COMB}}$$

(5) $${\text{R}}_{\text{US}}=\frac{\frac{1}{\text{c}}.\left|{\text{V}}_{\text{CS-US}}\right|}{{\text{α}}_{\text{CS}}+\frac{1}{\text{c}}.\left|{\text{V}}_{\text{CS-US}}\right|}{\text{V}}_{\text{COMB}}$$

(6) $$\text{r}1=\left(\frac{1}{\text{c}}.{\text{R}}_{\text{CS}}\times {\text{V}}_{\text{CS-r1}}\right)+\left(\frac{1}{\text{c}}.{\text{R}}_{\text{US}}\times {\text{V}}_{\text{US-r1}}\right)$$

**Fig. 3:**
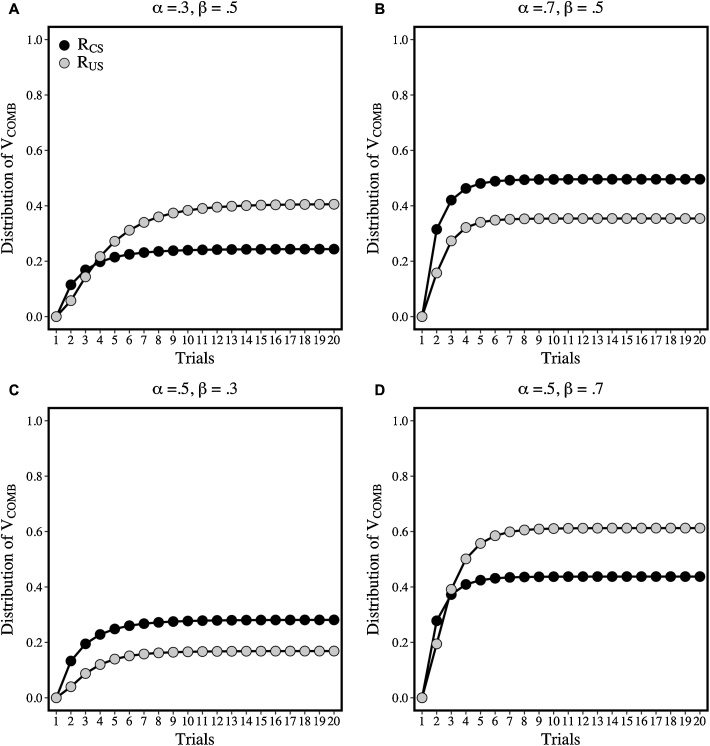
Simulations of the distribution of V_COMB_ into R_CS_ and R_US_ across 20 conditioning trials. R_CS_ (black symbols) and R_US_ (grey symbols) outputs were generated when the following values of α_CS_ and β_US_ were used in Eqs. (1), (2), (3), (4), (5). Panels A and B: α_CS_ was either .30 (A) or .70 (B) and β_US_ was fixed at .50. Panels C and D: α_CS_ was fixed at .50 and β_US_ was either .30 (C) or .70 (D). Adapted from: Honey, R.C., Dwyer, D.M., & Iliescu, A.F. (2020). HeiDI: A model for Pavlovian learning and performance with reciprocal associations. *Psychological Review* (in press).

Simulations confirm that changing the value of α_CS_ relative to β_US_ results in changes in R_CS_ relative R_US_: Fig. 3 shows that when α_CS_ > β_US_ then R_CS_ > R_US_ (see panels B and C), and when α_CS_ < β_US_ then R_CS_ < R_US_ (see panels A and D). If these differences are multiplied by the strengths of the links (see Fig. 2) between the CS and r1-r6 (e.g., V_CS-r1_) and the US and r1-6 (e.g., V_US-r1_), according to Eq. 6, then we have the basis for the translation of associative strength into different forms of behavior. We assume that the value of r1-r6, which takes units of V, is reflected in the vigor of their corresponding response forms.

**Fig. 4:**
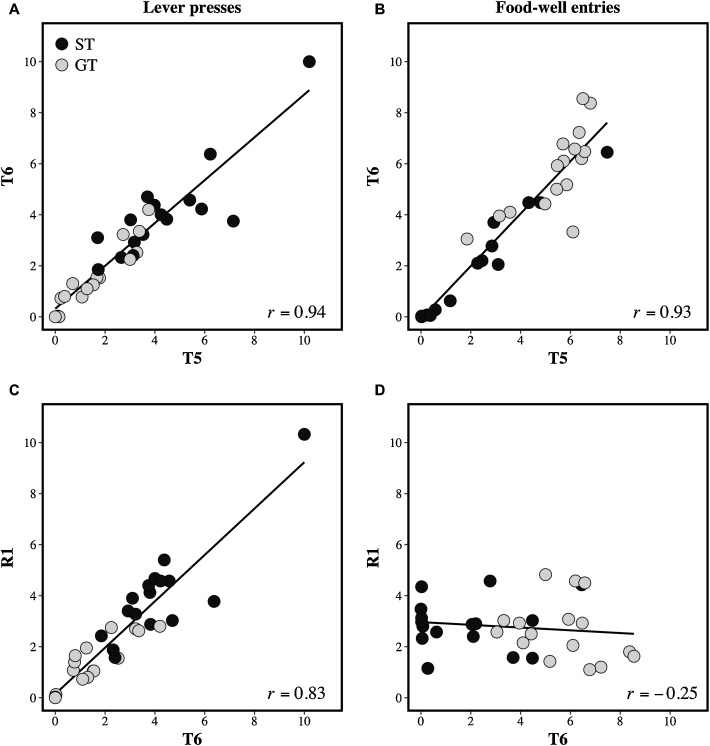
The upper panels show the relationship between the mean number of responses per reinforced trial for lever presses (left-hand panel) and for food-well entries (right-hand panel) on training blocks 5 (T5) and 6 (T6). The lower panels show the relationship between the final block of training (T6) and the first block on which the formerly reinforced lever was no longer reinforced (R1). The black symbols correspond to rats classified as sign-trackers (i.e., group ST) and the grey symbols to those classified as goal-trackers (i.e., group GT). Adapted from: Iliescu, A.F., Hall, J., Wilkinson, L., Dwyer, D.M., & Honey, R.C. (2018). The nature of phenotypic variation in Pavlovian conditioning. *Journal of Experimental Psychology*: *Animal Learning and Cognition*, *44*, 358-369.

HeiDI provides a simple associative analysis for a broad range of group-level phenomena that have proven to be a challenge over a protracted period, and does so without the need to appeal to some tendentious assumptions or additional (e.g., attentional) processes (see Honey et al., 2020a, 2020b). However, for the present purposes, it is most relevant to consider a series of findings that provide support for the analysis of individual differences in the form of responding that HeiDI provides; findings that are also beyond extant associative models. One finding that is consistent with the idea that β_US_ affects performance (through its impact on V_CS-US_) was reported by Patitucci et al. (Experiment 2, 2016). In this experiment, blocks of training in which rats received pairings of the presentation of a lever with the delivery of a sucrose reinforcer, were interposed with individual sessions where the affective responses of the rats to sucrose was assessed through an analysis of microstructure of the licking (see Dwyer, 2012). Patitucci et al. observed that individual differences in goal-tracking were a positively correlated with the affective value of sucrose, whereas sign-tracking had - if anything - a negative relationship with the affective value of sucrose (see also, Morrison, Bamkole, & Nicola, 2015). This is precisely what would be expected if the affective value of the US, as measured by the microstructure of licking, reflected β_US_. Patitucci et al. (Experiment 1, 2016) also observed that if the presentation of one lever was followed by one US (e.g., sucrose) while the presentation of a second lever was followed by a different US (e.g., food pellets) then the bias to either sign-tracking or goal-tracking on one lever was uncorrelated with the bias observed on the other lever. This observation is consistent with the interpretation that different USs have different β_US_ values across a group of rats. This interpretation received direct support from the observation that when the same US (food or sucrose) is paired with both levers then there was now a strong correlation between the biases shown during presentations of the two levers (Iliescu et al., 2018). Finally, HeiDI assumes that the current value of V_CS-US_ affects how V_COMB_ is distributed into R_CS_ and R_US_ (see Eqs. 4 and 5): when V_CS-US_ is low relative to α_CS_ then R_US_ < R_CS_. This assumption predicts that a procedure involving experimental extinction, which should result in a reduction in net V_CS-US_ while leaving α_CS_ the same, should result in the preferential distribution of V_COMB_ to the R_CS_ component (see Eq. 4) rather than to the R_US_ component (see Eq. 5); and thus sign-tracking being more resistant to extinction than goal-tracking. This is precisely what Iliescu et al. (Experiment 1, 2018) observed in an experiment in which rats first received a discrimination in which one lever was reinforced and a second was not, and then these contingencies were reversed (see also, Ahrens, Singer, Fitzpatrick, Morrow, & Robinson, 2016): The reduction in goal-tracking to the formerly reinforced lever was more rapid than the reduction in sign-tracking. This observation was apparent irrespective of whether the rats had an original bias to goal-track or sign-track (see also, Anselme, Robinson, & Berridge, 2012). The key results from the study by Iliescu et al. (2018) are depicted in Fig. 4.

The upper panels of Fig. 4 depict the relationships between lever presses on training blocks 5 and 6 (left panel) and between food-well entries on the same blocks (right panel). Each circle represents a given rat. The filled and open circles denote those classified as sign-trackers and goal-trackers, respectively, on the basis of their bias score on the final training block. There was a clear correlation between lever presses on blocks 5 and 6 and between food-well entries on these blocks. Moreover, the upper panels indicate that the rats designated sign-trackers showed higher levels of lever presses than those designated as goal-trackers, and rats designated goal-trackers showed higher levers of food-well entries than those designated sign-trackers. The lower panels depict the relationships between the two forms of responding on the final block of training and the first block in which the contingencies were changed (i.e., reversed). It is clear that while lever pressing remained relatively unchanged by this manipulation, goal-tracking no longer reflected the previous contingencies. In fact, over the course of reversal training, goal-tracking came to rapidly match the changed contingencies while sign-tracking changed less rapidly (see Iliescu et al., 2018).

**Fig. 5:**
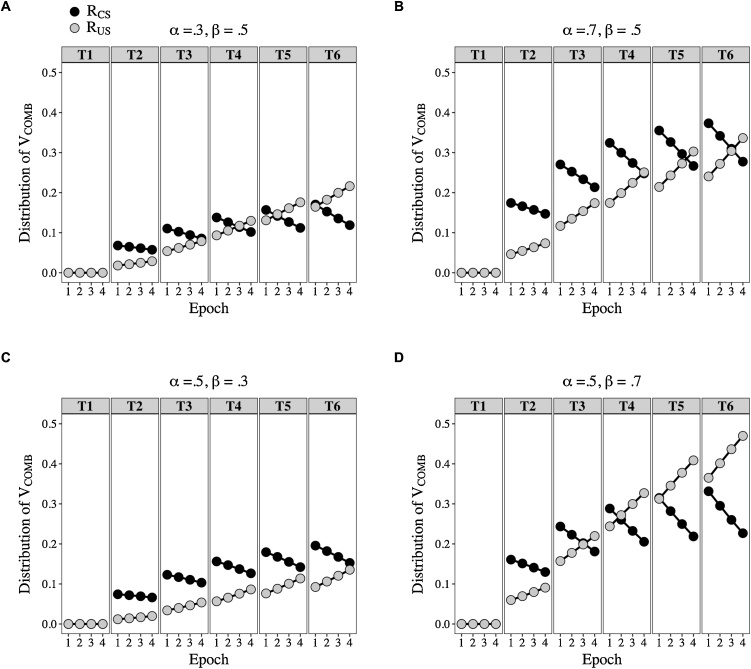
Simulations of R_CS_ and R_US_ across training blocks (T1-T6) and trial epoch (1-4). The V_COMB_ outputs used in Eqs. 4 and 5 to calculate R_CS_ (black symbols) and R_US_ (grey symbols) were generated using Eqs. (1), (2), (3). In panels A and B, at the start of a CS presentation α_CS_ was either .3 (A) or .7 (B) and β_US_ was fixed at .5; and in panels C and D, at the start of the CS presentation α_CS_ was fixed at .5 and β_US_ was either .3 (C) or .7 (D). Across the 4 epochs of a trial, the value of α_CS_ was subject to exponential decay (α_CS_(1 – 0.10)^2^) and was reset at the start of each trial. The terminal values of α_CS_ were used in Eqs. (1), (2), (3), while the (within-trial) decaying values were used in Eqs. 4 and 5. Adapted from: Iliescu, A.F., Dwyer, D.M., & Honey, R.C. (2020). Individual differences in the nature of conditioned behavior across a conditioned stimulus: Development and application of a model. *Journal of Experimental Psychology*: *Animal Learning and Cognition* (in press).

**Fig. 6:**
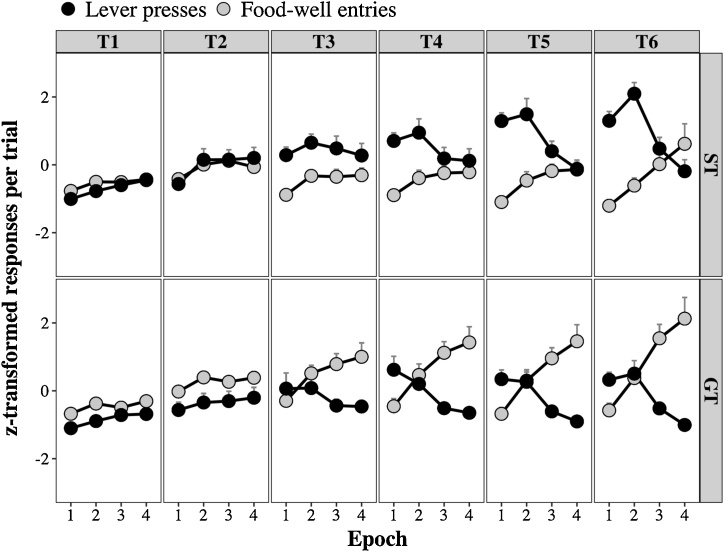
Mean (+SEM) z-transformed lever presses (black symbols) and food-well entries (grey symbols) during reinforced lever presentations in groups ST (upper panels) and GT (lower panels). The results from the 10-s lever presentations are broken down into 4 successive 2.5-s epochs for each of the 6 blocks of training (T1-T6). Adapted from: Iliescu, A.F., Dwyer, D.M., & Honey, R.C. (2020). Individual differences in the nature of conditioned behavior across a conditioned stimulus: Development and application of a model. *Journal of Experimental Psychology*: *Animal Learning and Cognition* (in press).

The evidence presented thus far has implicated the perceived salience of the US in the distribution of US-oriented and CS-oriented behavior. HeiDI also supposes that the perceived salience of the CS should have an effect: a CS with higher perceived salience should result in more CS-oriented and less US-oriented behavior. We do not have any direct evidence that bears on this prediction. However, if one assumed that the perceived salience of the CS is high at the outset of a stimulus and declines across its duration (e.g., due to habituation), then this should be evident as a reduction in sign-tracking and an increase in goal-tracking across the duration of a CS. That is, a plausible additional assumption predicts that while some indices of conditioning should decline across a CS, others should increase (i.e., show inhibition of delay; Pavlov, 1927). It is worth noting that a similar idea was briefly entertained by Mackintosh (p. 62, 1974; see also, Pavlov, 1927, p. 104; Staddon, 2005; Staddon & Higa, 1999; Wagner, 1981), who argued that a decay process might enable the early and later parts of a CS to be discriminated, and thereby provide a basis for inhibition of delay. In any case, formal simulations of HeiDI together with an analysis of archival data confirmed the accuracy of the predictions identified above (see Fig. 5, Fig. 6; Iliescu, Dwyer, & Honey, 2020).

The simulations were conducted with the α_CS_ values set from the outset of the CS at either .30 or .70 and β_US_ fixed at .50 (panels A ad B of Fig. 5), or with the α_CS_ value set from the outset of the CS at .50 and β_US_ set to .30 or .70 (panels C and D of Fig. 5). Across the 4 epochs of each trial, these α_CS_ values were subject to exponential decay: α_CS_(1 – 0.10)^2^. The α_CS_ value in the fourth epoch was used to calculate the change in associative strength in Eqs. 1 and 2. R_CS_ and R_US_ were calculated by entering the decaying value of α_CS_ into Eqs. 4 and 5; and α_CS_ was reset to its starting value after a given trial. It is clear that irrespective of whether the values for the critical parameters, α_CS_ and β_US_, were set to generate higher values of R_US_ relative to R_CS_ (panels A and D) or higher values of R_CS_ relative to R_US_ (panels B an C), R_US_ increased across epochs within a trial while R_CS_ decreased.

Fig. 6 shows how the lever presses and food-well entries change across training blocks (T1-T6) and across four 2.5-s epochs within the 10-s trials, with the upper panes depicting a group of rats designated sign-trackers (group ST) and the lower panes depicting a group of rats designated goal-trackers (group GT). z-transformed scores were used to put the different responses (lever presses and food-well entries) on the same scale. Inspection of Fig. 6 shows that the lever-pressing bias in group ST was most evident at the start of trials, whereas food-well entry bias in group GT was most evident at the end of the trials (see also, Derman, Schneider, Juarez, & Delamater, 2018; but see, Lee et al., 2018). These patterns of results match those predicted by HeiDI.

## 3 General discussion

Individual variation in the form of Pavlovian conditioned behavior has been largely neglected by general process models of associative learning. Here, we have illustrated how one model, HeiDI, provides a simple analysis for such variations. The analysis relies on a novel specification of the associative structures that underpin learning and performance (see Fig. 2), and the rules governing how changes occur within those structures (Eqs. 1 and 2). These rules have two free parameters, α_CS_ and β_US_, which are aligned to the perceived salience of the CS and US, respectively. These parameters are assumed to be fixed for a given rat, but to vary across a set of rats. Variation in the two parameters thereby provides the basis for individual differences in the vigor and form of conditioned behavior. Returning to Watson (1924), we now have a general process model of Pavlovian conditioning that provides a basis for both quantitative and qualitative individual differences in acquired behavior – changes that emerge as a consequence of giving the same training to a group of rats. What is more, this model also provides an analysis for group-level phenomena that have proven resistant to alternative associative models (see Honey et al., 2020a, 2020b). While HeiDI was developed in the context of findings from a model system (i.e., sign- and goal-tracking in rats), it has very broad explanatory power, only some of which derives from its adaptation of the pooled error term proposed by Rescorla and Wagner (1972).

The analysis offered by HeiDI for individual differences in Pavlovian conditioned responding rests on a single learning process – the development of reciprocal associations between the CS ad US – the behavioral sequelae of which are influenced by the relative perceived saliences of the CS (i.e., α_CS_) and the US (i.e., β_US_; as given by V_CS-US_). However, others have explicitly argued that sign-tracking and goal-tracking, in particular, originate in two process of learning: with sign-tracking reflecting the fact that a lever CS gains incentive salience through its association with the US, while goal-tracking reflects the predictive value of the lever CS (see Berridge & Robinson, 2016; see also, Flagel, Akil, & Robinson, 2009; Lesaint, Sigaud, Flagel, Robinson, & Khamassi, 2014). Similarly, it has been suggested that while sign-tracking reflects a stimulus-response association (i.e., a lever-response association), goal-tracking reflects a stimulus-stimulus association (i.e., lever-outcome association; see Iliescu et al., 2018; Patitucci et al., 2016). These less parsimonious, dual-process accounts predict some of the results that are consistent with HeiDI, particularly the fact that sign-tracking is more resistant to extinction than is goal-tracking (e.g., Iliescu et al., 2018), and the related fact that a partial reinforcement schedule (or uncertainty) maintains higher levels of sign-tracking than does a continuous reinforcement schedule (see Robinson, Anselme, Fischer, & Berridge, 2014). However, these accounts are rather less consistent with other aspects of the behavioral results that we have summarized here, which provide support for HeiDI. For example, they provide no very clear basis for the observation that the sign-tracking phenotype is most evident at the outset of a CS while the goal-tracking phenotype is most evident at the end of the stimulus (see Iliescu et al., 2020). Moreover, and as we have already noted, HeiDI provides an analysis of a very broad range of group-level phenomena that are simply beyond the scope of models developed in the context of one model system: Autoshaping in the rat.^1^

If we accept the analysis provided by HeiDI for variation in the vigor and form of Pavlovian conditioned behavior, then a natural question is what are the origins of differences in the perceived salience of the CS (i.e., α_CS_) and US (i.e., β_US_)? The simple answer to this question is that we do not know. But, one issue that might well constrain our capacity to address this question at behavioral, computation and neural levels is the fact that the sign-tracking and goal-tracking responses are (necessarily) quite different in a standard rat autoshaping procedure: A lever enables a set of responses that is simply very different from the set of responses directed towards the food well. This fact makes it easy to identify the two forms of responding, but it does not allow their ready comparison. To circumvent this issue, it would be advantageous to develop procedures where the sign-tracking and goal-tracking responses are measured (more) equivalently. For example, one could have adjacent food wells in which the (internal) illumination of one food well signals the impending arrival of food in the other food well. In this way, the measures of sign- and goal-tracking would be much more similar: Sign-tracking would be evident as entering the illuminated food well and goal-tracking by entering the well in which food was delivered. It remains to be seen whether this approach is a viable one: Will it yield individual differences in the form of conditioned responding that are evident in the conventional rat autoshaping procedure.

To summarize: The development of HeiDI was inspired by the marked quantitative but especially qualitative individual differences in the form of conditioned responding observed in a Pavlovian conditioning procedure. These differences have not been dealt with by extant general process models of Pavlovian conditioning, for which associative strength (or some other unitary construct) is assumed to have a monotonic relationship to conditioned behavior (e.g., Mackintosh, 1975; Pearce & Hall, 1980; Rescorla & Wagner, 1972; see also, Gallistel & Gibbon, 2000; Stout & Miller, 2007). It should be noted that the rat autoshaping procedure in which the qualitative differences are observed is not one that is routine: While the appetitive US is standard, the lever CS is not. However, many of the behavioral phenomena that provide converging support for HeiDI from this procedure have clear counterparts in other conditioning procedures (see Honey et al., 2020a, 2020b). Under these conditions, it seems entirely appropriate that HeiDI is formulated as a general process model, providing a relatively simple analysis for an extensive array of findings.

## Author note

This research was partly conducted when A.F.I. was supported by a School of Psychology PhD studentship, and supervised by R.C.H and D.M.D; and the development of HeiDI was also supported by a grant awarded to R.C.H. and D.M.D. by the 10.13039/501100000268BBSRC (UK; BB/T004339/1). All three authors contributed to the ideas presented in this manuscript and to its preparation.

We dedicate this article to our dear friend and unique colleague, William J. Macken (Bill), who passed in February 2020. Correspondence about this article should be addressed to: R.C. Honey; email: honey@cardiff.ac.uk.

## Author statement

All three authors contributed to the ideas presented in this manuscript, and to its preparation for publication.

## Footnotes

1 It should be apparent, however, that there are other potential sources of variation within HeiDI that could also contribute to differences in the form of responding: The pre-existing differences in the connections between the CS, US and r1-r6; and the obvious potential for these connections to change (see Fig. 2; Honey, Dwyer, & Iliescu, 2020).
